# Decreased intranuclear cardiac troponin I impairs cardiac autophagy through FOS/ATG5 in ageing hearts

**DOI:** 10.1111/jcmm.18357

**Published:** 2024-04-29

**Authors:** Rui Min Liu, Shan Huang, Di Hu, Lingjuan Liu, Hui Chao Sun, Jie Tian, Bo Pan

**Affiliations:** ^1^ Department of Pediatric Cardiology National Clinical Key Cardiovascular Specialty Chongqing China; ^2^ Ministry of Education Key Laboratory of Child Development and Disorders Chongqing China; ^3^ National Clinical Research Center for Child Health and Disorders Chongqing China; ^4^ China International Science and Technology Cooperation Base of Child Development and Critical Disorders Chongqing China; ^5^ Key Laboratory of Children's Important Organ Development and Diseases of Chongqing Municipal Health Commission Chongqing China; ^6^ Children's Hospital of Chongqing Medical University Chongqing China; ^7^ Maternal‐Fetal Medicine Center in Fetal Heart Disease, Capital Medical University Beijing Anzhen Hospital Beijing China; ^8^ Department of Otorhinolaryngology Children's Hospital of Chongqing Medical University Chongqing China

**Keywords:** ageing, autophagy, FOS, intranuclear cTnI

## Abstract

In our previous study, intranuclear cardiac troponin I (cTnI) may function as a co‐factor of Yin Yang 1(YY1). Here, we aimed to explore the role of intranuclear cTnI in ageing hearts. Nuclear translocation of cTnI was demonstrated using Western blot and immunofluorescence. The potential nuclear localization sequences (NLSs) of cTnI were predicted by a web server and then verified in 293T cells by putative NLS‐eGFP‐GST and NLS‐mutant transfection. The ratio of Nuclear cTnI/ Total cTnI (Nu/T) decreased significantly in ageing hearts, accompanied with ATG5‐decline‐related impaired cardiac autophagy. RNA sequencing was performed in cTnI knockout hearts. The differential expressed genes (DEGs) were analysed by overlapping with YY1 ChIP‐sequencing data. cTnI gain and loss experiments in vitro determined those filtered DEGs' expression levels. A strong correlation was found between expression patterns cTnI and FOS. Using ChIP‐q‐PCR, we demonstrated that specific binding DNA sequences of cTnI were enriched in the FOS promoter −299 to −157 region. It was further verified that pcDNA3.1 (−)‐cTnI could increase the promoter activity of FOS by using luciferase report assay. At last, we found that FOS can regulate the ATG5 (autophagy‐related gene 5) gene by using a luciferase report assay. Taken together, our results indicate that decreased intranuclear cTnI in ageing hearts may cause impaired cardiac autophagy through the FOS/ATG5 pathway.

## 1 INTRODUCTION

The population of ageing has already become a global problem and has led to a remarkable increase in the prevalence of age‐associated diseases, especially cardiovascular diseases (CVDs).^1^ Understanding the mechanisms of cardiac ageing will help reduce the incidence of CVD and provide potential interventions and treatments for cardiac ageing. Accumulating data suggest that multiple complex pathways involved in cardiac ageing, such as oxidative stress, autophagy and mTOR signalling.^2^ Autophagy, a highly conserved evolutionarily process, is essential for the maintenance of cellular homeostasis through the degradation of long‐lived proteins and functionally redundant or damaged intracellular organelles in lysosomes.^3^, ^4^ Cardiac autophagy is decreased with age, and dysfunctional mitochondria and misfolded proteins accumulate in the ageing heart.^5^ Therefore, autophagy represents a promising intervention target for ageing‐induced cardiac dysfunction. For example, the autophagy‐related gene ATG5 cardiac‐specific deletion in the mouse induces age‐related cardiac dysfunction.^6^ In contrast, overexpression of ATG5 in mice activates autophagy and extends lifespan.^4^

The FOS gene family consists of four members: FOS, FOSB, FOSL1 and FOSL2. These genes encode leucine zipper proteins that can dimerize with proteins of the JUN family, thereby forming the transcription factor complex AP‐1.^7^ As such, the FOS proteins have been implicated as regulators of cell proliferation, differentiation and transformation.^7^, ^8^ In some cases, expression of the FOS gene has also been associated with apoptotic cell death and autophagy.^9^

In others and our previous studies, nuclear translocation of cTnI was found in mouse, human fetuses and rat heart tissues.^10^, ^11^, ^12^, ^13^, ^14^ Moreover, intranuclear cTnI might regulate the Apt2a2 transcription level through binding with a classic transcription factor YY1, affecting cardiomyocyte contraction and relaxation.^14^ However, the potential nuclear localization sequences of cTnI, and more importantly, the role of intranuclear cTnI in cardiac pathological conditions remain unknown and are worth substantial attention. We first found an interesting phenomenon: the proportion of intranuclear cTnI to total cTnI (Nu/T cTnI) in ageing mouse hearts decreased significantly compared with that in young adult mouse hearts. Moreover, cardiac autophagy levels in cTnI knock‐out heterogynous (cTnI^−/+^) mice were reduced considerably. Therefore, the objective of the present study was to determine the role of intranuclear cTnI in cardiac ageing. RNA sequencing was performed in hearts of cTnI knock‐out mice. As cTnI might function as a co‐factor of YY1, we then analysed DEGs by overlapping with YY1 ChIP‐sequencing data. Several ageing‐related genes, such as s100a8, FOS, FOSb and Erg1, were filtered by overlapping analysis. By cTnI gain and loss in vitro studies, a strong expression correlation between cTnI and FOS was found. By using ChIP‐q‐PCR and luciferase report assays, it was demonstrated that cTnI plasmid can enhance the promoter activity of FOS. At last, FOS was shown to improve the transcriptional activity of ATG5 through dural luciferase report assay. Our data indicate that decreased intranuclear cTnI may reduce cardiac autophagy levels by regulating FOS/ATG5 expression.

## 2 MATERIALS AND METHODS

### 2.1 Animals

Four‐week‐old, 3‐month‐old and 24‐month‐old C57BL/6 male mice were purchased from the Experimental Animal Center of Chongqing Medical University (Chongqing, China). Heterozygous 12 month‐old cTnI^+/−^ mutant mice were described previously,^14^, ^15^ and kindly provided by Professor Huang (Florida Atlantic University) as a gift. All experimental procedures involving animals were approved by the Animal Care and Use Committee of Chongqing Medical University (2020‐1102). Animal experiments were performed to conform to the NIH guidelines (Guide for the Care and Use of Laboratory Animals). All animals were euthanized by administration of pentobarbiturate (100–120 mg/kg).

### 2.2 Neonatal mice ventricular myocytes (NMVMs) isolation and culture

As previously described,^14^ NRVMs were isolated from day 1 mouse pups using enzymatic digestion. Briefly, the cells were digested by 0.05% collagenase II, centrifuged by density gradient and collected to seed at 1 × 10^6^ cells/well in a 6‐well plate. The cells were grown in F12/DMEM (1:1) containing 10% FBS, penicillin and streptomycin (50 U/mL), 5‐Fluorouracil(0.1 g/L) at 37°C in humid air containing 5% CO2. After 48 h of adhering, the culture medium changed every day.

### 2.3 NLS prediction and fusion protein construction

We employed a program, PSORTII (psort.nibb.ac.jp), designed to predict the protein sorting signals and the localization sites to predict the potential NLSs of cTnI. To generate cTnI deletion mutants for expressing fusion protein, we inserted PCR‐generated cDNA fragments encoding cTnI amino acids 1–212, 1–170, 1–137, 1–47, 46–137, 137–170 and 170–212 into the PCDNA3.1‐GFP‐GST expression plasmid. We generated WT cTnI by cloning the full‐length cTnI (amino acids 1–212) into the pcDNA3.1 expression vector. The cTnI mutation at R21/R22 (lysine or arginine residue) in predicted NLS (amino acids 16–22) of the cTnI were created by using a Quik change site‐directed mutagenesis kit (Stratagene), pcDNA3.1‐ cTnI was employed as templates. Plasmid PCDNA3.1 was kind gift from Dr Xi Li (Biology Science Institutes, Chongqing Medical University, China).

### 2.4 RNA‐sequencing and ChIP‐sequencing overlapping data analysis

RNA from the hearts of 15‐ to 17‐day postnatal mice were prepared for gene expression profiling (3 biological replicates for each group). The KO and WT samples were sequenced on the cBot Cluster Generation System using TruSeq PE Cluster Kit v3‐cBot‐HS (Illumia). The sequencing data were filtered with SOAPnuke (v1.5.2) by removing reads containing sequencing adapter, reads whose low‐quality base ratio (base quality less than or equal to 5) is more than 20% and reads whose unknown base (‘N’ base) ratio is more than 5%. The resultant clean reads were obtained, stored in FASTQ format and mapped to the reference genome GRCm38.p6 using HISAT2 (v2.0.4). Bowtie2 (v2.2.5) was applied to align the clean reads to the reference coding gene set, and the expression level of genes was calculated with RSEM (v1.2.12). The differential expression analysis was performed using the DESeq2 (v1.4.5). GO enrichment analysis of differentially expressed genes was achieved by cluster Profiler (3.4.4) software, which was corrected for gene length bias. GO terms with corrected *p* values of less than 0.05 were considered to be significantly enriched by differentially expressed genes. YY1 chromatin immunoprecipitation sequencing data were downloaded from Cistrome DB‐57271. RNA‐seq data and ChIP‐seq data were overlapped and analysed.

### 2.5 Immunofluorescence

NMVMs cells were seeded in 12‐well plates containing cover glasses (WHB scientific, #12‐CS). Cells were fixed with 4% paraformaldehyde (15 min at room temperature), and their membrane were permeabilized at room temperature with 0.5% Triton X‐100 in phosphate‐buffered saline (PBS) buffer for 20 min. After three washes with PBS, cells were blocked with 4% BSA (Solarbio, #SW3015) blocking solution at room temperature for 30 min. Cells were incubated with the primary antibody (1:200) overnight at 4°C. Then, cells were incubated with the fluorescent secondary antibodies at room temperature for 1 h after three times PBST washes. Next, cells were counterstained with DAPI (Beyotime, #C1006) for 5 min to visualize nuclei. Immunofluorescence images were taken by using confocal microscopy (Nikon C2 plus, Japan).

### 2.6 Total RNA extraction and quantitative RT‐PCR

The total RNA of all samples was extracted with the Tissue RNA Extraction Kit (Bioflux, Beijing, China). cDNA was obtained by using the RevertAid First Strand cDNA Synthesis Kit (#No, RR047A, TaKaRa, Japan). The cDNA was amplified with SYBR® Green Master Mix kit (#204054, Qiagen, Germany). Analyses of relative mRNA expression were determined using the 2^−ΔΔCt^ method, normalized to the expression of housekeeping gene. Primers for the genes were synthesized by Life Technologies Corporation (Shanghai, China). Sequences of PCR primers used in this study are listed in Table S1.

### 2.7 Western blot analyses

Western blot was carried out as previously described.^14^, ^16^ Briefly, protein lysate samples were prepared from heart tissues in RIPA buffer with proteinase inhibitors. Lysate samples (15–20 μg total protein for each) were separated by 10% or 12% SDS‐PAGE and electrophoretically transferred to PVDF membranes. cTnI protein was probed with mouse anti‐cTnI antibodies (Santa, #sc‐133117). HLC3B protein was probed with Rabbit anti‐LC3B antibodies (CST, #12741), p62 protein was probed with Rabbit anti‐p62 antibodies (CST, #23214), c‐Fos was probed with Rabbit anti‐c‐Fos antibodies (CST, 2250). GAPDH (Proteintech, #60004‐1), β‐actin (Proteintech, #81115‐1), and Laminb1 (CST, #13435) antibodies served as loading controls for different protein analyses. The densitometric analysis was performed using ImageJ software.

### 2.8 RNA interference (RNAi) assays

Synthetic siRNA oligonucleotides are specific for TNNI3 (5′‐GAAGAUCUAUGACCUCCGUTT‐3′), which was synthesized by GenePharma (Shanghai, China). Stealth RNAi Negative Control Duplexes by GenePharma were used as negative controls. According to the manufacturer's instructions, NMVMs were transiently transfected with TNNI3 siRNA or negative control (NC) siRNA using GP‐transfection‐Mate (GenePharma, Shanghai, China).

### 2.9 Overexpression of TNNI3

GV314Ad‐TNNI3 and GV314AD‐GFP adenoviral vectors were purchased from Gene‐Chem Technology Co., Ltd. (Shanghai, China). NRVMs were plated onto slides in six‐well plates and allowed to reach 50%–70% confluence at the time of transfection. The GV314Ad‐GFP adenovirus was used as a control (negative control, NC). Adenoviral infection was performed according to the manufacturer's instructions. NRVMs were incubated in a growth medium with the adenoviruses at a multiplicity of infection of 40 for 12 h at 37°C and were then grown in a new medium for another 60 h at 37°C.

### 2.10 ChIP‐qPCR

Following the previous steps, purified DNA was subjected to quantitative PCR using primers specific to the FOS promoters. The primers (mouse) of involved DNA sequence were designed as below:

Site1: 5′‐ GGCGCTGTGTTGCTGTAAAC ‐3′ (forward primer)
5′‐ CGGATGGATCTTTAGGGGCG ‐3′ (reverse primer)
Site2: 5′‐ AGGGCAAGTAGGGGTGTGTT ‐3′ (forward primer)
5′‐ GGGGACGGGAAGAAAGGTTC ‐3′ (reverse primer)
Site3: 5′‐ AACATACGACCCCTTCAGGC ‐3′ (forward primer)
5′‐ CACCTACTCCCCGACCCTTA ‐3′ (reverse primer)
Site4: 5′‐ GGAACCGGGTCCACATTGA ‐3′ (forward primer)
5′‐ AGGGATTGACGGGAACAGC ‐3′ (reverse primer)
Site5: 5′‐ AATCCTACACGCGGAAGGTC ‐3′ (forward primer)
5′‐ GTCTTGGCATACATCTTTCACCT ‐3′ (reverse primer)
Site6: 5′‐ GCGTAGAGTTGACGACAGAG ‐3′ (forward primer)
5′‐ ACTTCCTACGTCACTGGGC ‐3′ (reverse primer).

### 2.11 Dural luciferase report assay

−299 to −157 bp promoter regions of mouse FOS, which were detected by ChIP‐Seq, were highly enriched with cTnI. In addition, these 2000 bp bases were amplified via PCR from the genomic DNA of C57BL/6 mice and were cloned into PGL3‐FOS‐WT. 5′‐CCAT‐3′ were interchanged to 5′‐AACC‐3′ (−299 to −157 bp), mutation vectors of pGL3‐ FOS‐MUT were constructed. JASPAR website was used to predict the specific binding sequence of the transcription factor FOS gene in the ATG5 promoter region. The fragments relative to the transcription start site of the ATG5 genomic sequence (−2000 bp to 0) were cloned into PGL3‐ATG5‐WT; the mutant plasmid was constructed by 5′‐GCTC‐3′ interchanged to 5′‐AACA‐3′. TNNI3 and FOS expression vectors were prepared by cloning wild‐type mouse TNNI3 cDNA (636 bp) and FOS cDNA (1143 bp) into a pcDNA3.1 (−) vector. 293T cells were plated in 24‐well plate reached to 90%–95% confluent. PGL3‐FOS‐WT/PGL3‐FOS‐MUT/PGL3‐ATG5‐WT/PGL3‐ATG5‐MUT promoter fused firefly luciferase (300 ng/well), TK‐renilla luciferase (2 ng/well) and pcDNA3.1(−)‐TNNI3 (300 ng/well) or pcDNA3.1(−)‐FOS were transfected by Lipofectamine 3000 (NO. L3000015, Invitrogen, USA). Luciferase activity was measured via a luciferase reporter assay (E1910, Promega, USA), with firefly luciferase activity normalized to renilla activity.

### 2.12 Statistics

Three biological replicates were used for mRNA‐seq experiments. All other experiments have been repeated at least three times. Values are expressed as the mean ± SD. Student's *t*‐test was performed for paired analysis (two‐tailed, adjusted for multiple comparisons).

## 3 RESULTS

### 3.1 Nuclear localization of cardiac troponin I

**FIGURE 1:**
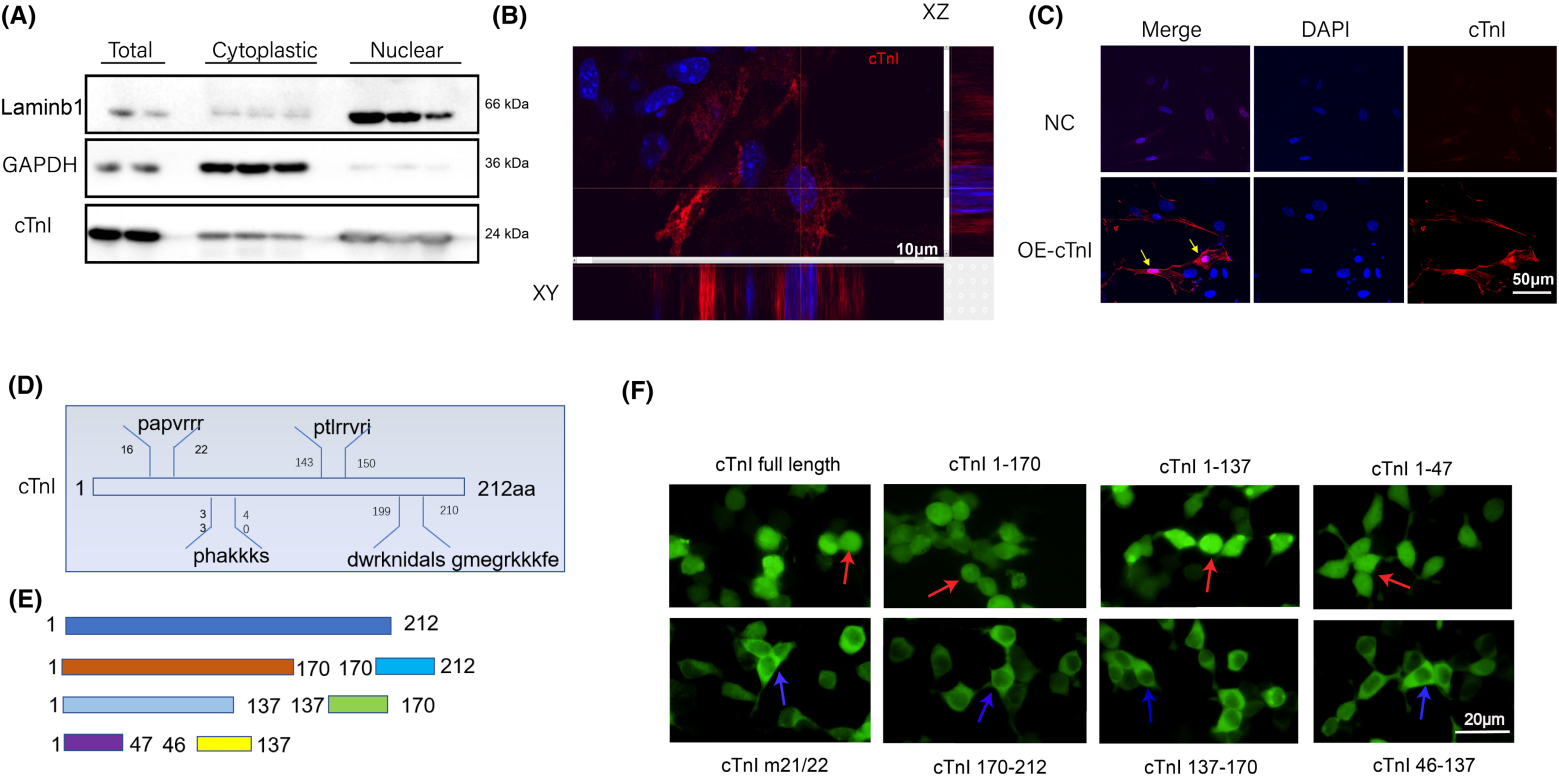
Nuclear localization of cardiac troponin I. (A) Western blot to detect cTnI protein expression in cytoplasm and nuclei, GAPDH and Laminb1 was selected as cytoplastic and nuclear control, respectively. (B) Confocal laser scanning microscope to detect cTnI localization through three‐dimensional reconstruction of neonatal mouse cardiomyocytes. (C) Fluorescence microscope to detect OE‐cTnI in neonatal mouse cardiomyocytes. (D) Candidate NLSs of cTnI by PSORTII. (E) Schematic diagram of cTnI fusion proteins. (F) cTnI fusion proteins localization in 293T cells, red arrows indicate cTnI locate in nuclear, and blue arrows indicate negative fluoresce of 293T nuclear. cTnI m21/22 stands for 21st and 22nd aa mutation of cTnI. Scale bar = 10 μm (B), 50 μm (C), 20 μm (F).

To verify the nuclear location of cTnI, we first detected cTnI protein expression in adult mouse hearts. As illustrated in Figure 1A, a certain amount of cTnI is expressed in the nucleus in adult mice. Using a confocal laser scanning microscope, three‐dimensional reconstruction of neonatal mouse cardiomyocyte images showed that cTnI can be expressed in the nucleus (Figure 1B). As an isoform switch of TnI, from ssTnI to cTnI during cardiomyocyte maturation after birth, cTnI is not the dominant isoform in neonatal cardiomyocytes,^16^ it was overexpressed by using an adenoviral plasmid. cTnI nuclear localization was also found in cTnI overexpressed cardiomyocytes (Figure 1C). These parts of the results were consistent with our previous findings. We then asked if cTnI has nuclear localization sequences (NLSs). Four potential NLSs of cTnI were predicted by a web server (Figure 1D). PCR‐generated cDNA fragments encoding cTnI amino acid fragments, 1–212, 1–170, 1–137, 1–47, 46–137, 137–170 and 170–212 were inserted into PCDNA3.1‐GFP‐GST expression plasmid, respectively (Figure 1E). As shown in Figure 1F, a fusion protein that contains 16‐22aa could be detected in the nuclear, and full‐length cTnI with mutant 21st and 22nd amino acids could not be detected in the nucleus (Figure 1F). Those data suggested that cTnI is located in the nucleus, and NLS of cTnI might be located on segments from 16 to 22 amino acids.

### 3.2 Intranuclear cTnI and cardiac autophagy levels decreased in ageing hearts

**FIGURE 2:**
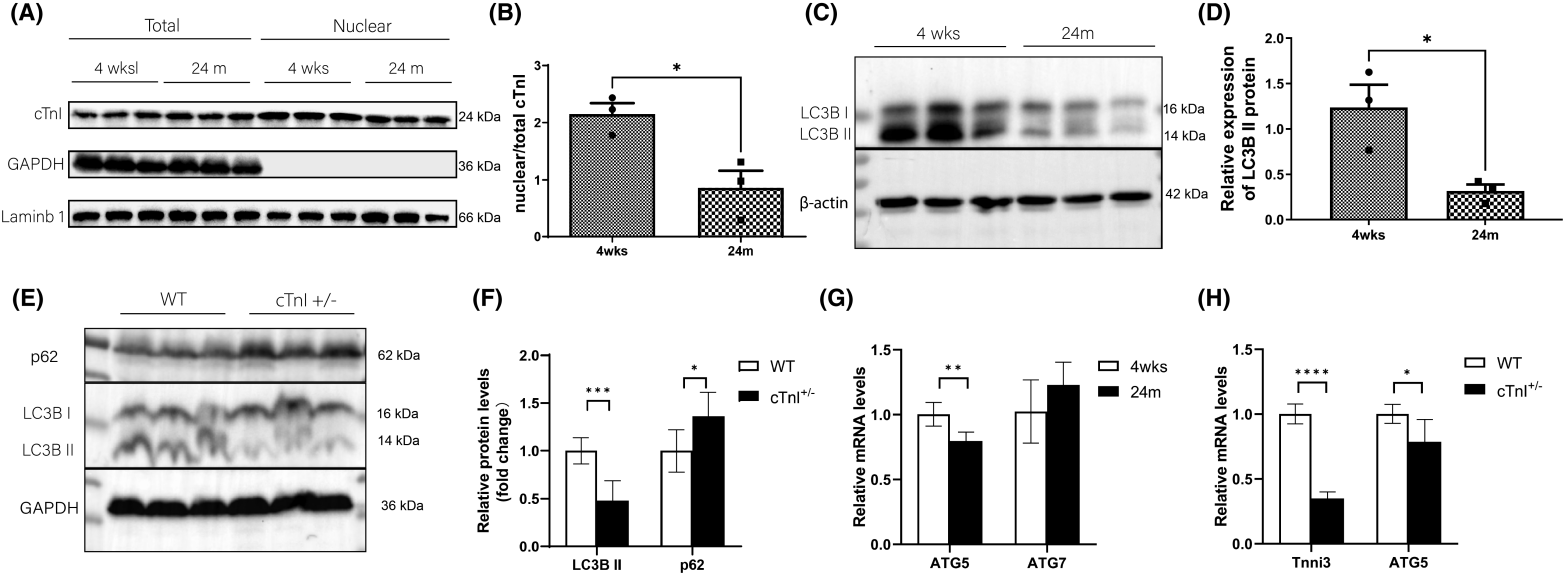
Intranuclear cTnI and cardiac autophagy levels decreased in ageing hearts. (A, B) Intranuclear and total cTnI protein levels detection, *n* = 3, 4 weeks: 4 weeks old, 24 months: 24 months old. (C, D) LC3BI and LC3BII protein levels detection in 4 weeks and 24 months mouse hearts, *n* = 3, β‐acin as loading control. (E, F) LC3BI, LC3BII and p62 protein levels in WT (12‐month) and cTnI^+/−^ (12‐month) mouse hearts, *n* = 3, GAPDH as loading control. (G) mRNA levels of ATG5 and ATG7 in 4 weeks and 24 months mouse hearts, *n* = 5. (H) Tnni3 and ATG5 mRNA levels in WT (12 month) and cTnI^+/−^ (12 month) mouse hearts. *n* = 3. *p < 0.05; **p < 0.01; ***p < 0.001; ****p < 0.0001. Values are expressed as mean ± SD.

We then asked about the role of intranuclear cTnI. Intranuclear cTnI levels were examined in ageing hearts (Figure S1, Figure 2). Interestingly, the percentage of nuclear/total cTnI decreased significantly in ageing hearts (Figure 2A,B). Meanwhile, our data and other reports both indicated that cardiac autophagy levels were reduced in ageing hearts, as autophagy markers LC3BII and ATG5 statistically declined in 24 months mouse hearts (Figure 2C,D,G). Moreover, cardiac autophagy was also impaired in cTnI knockout heterozygous (cTnI^+/−^) mouse hearts. As it was found that levels of LC3BII and ATG5 decreased, p62 protein levels increased (Figure 2E,F,H). These data suggested that decreased nuclear/total cTnI might associated with impaired cardiac autophagy in ageing hearts.

### 3.3 Overlapping of RNA‐sequencing and ChIP‐sequencing data, cTnI gain and loss in vitro

**FIGURE 3:**
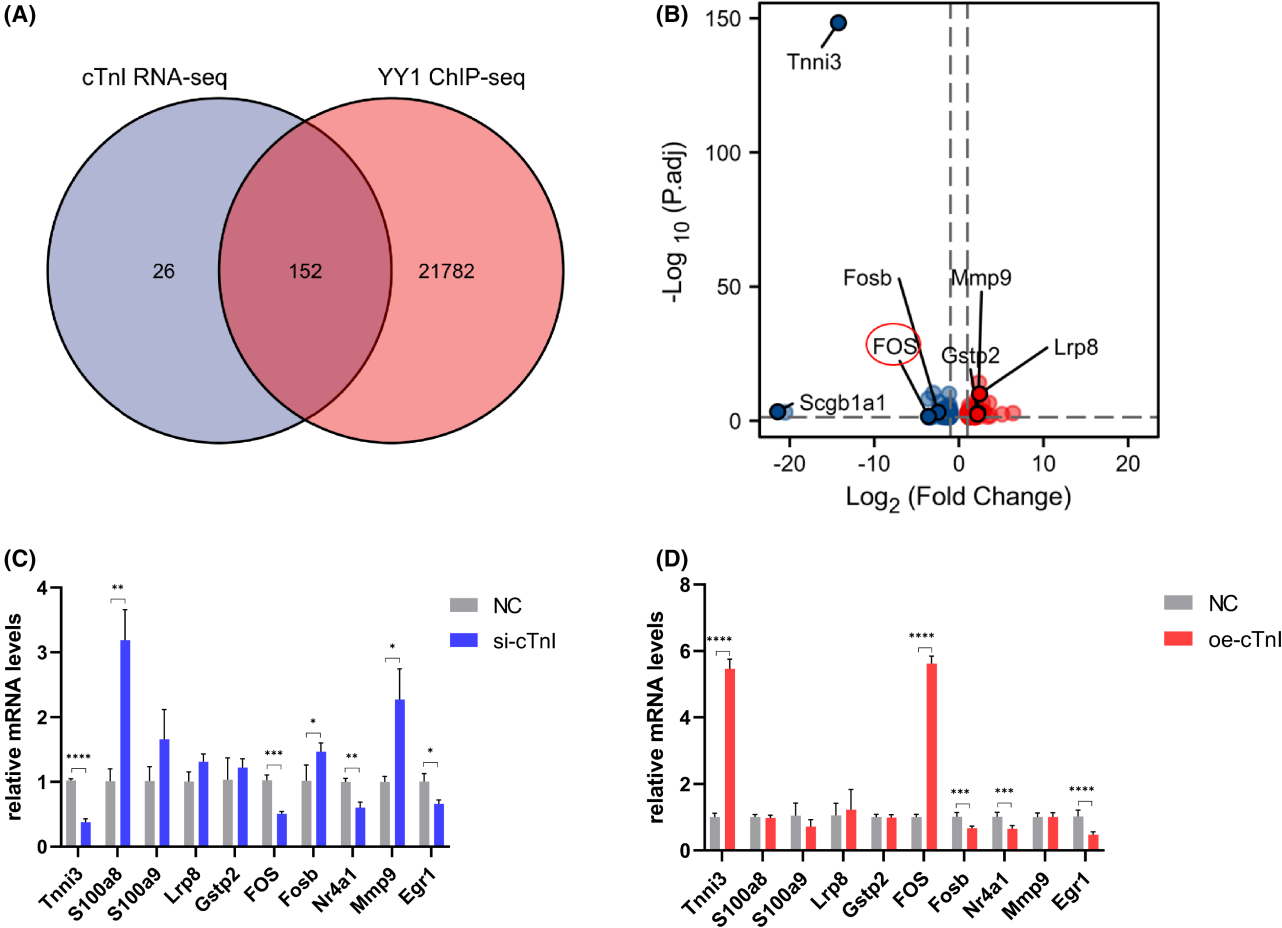
Overlapping of RNA‐sequencing and ChIP‐sequencing Data, cTnI gain and loss in vitro. (A) Overlapping weighted analysis of cTnI knock out RNA‐seq data and YY1 ChIP‐seq data. (B) Volcano diagram of overlapping genges. (C) Candidate genes mRNA expression detection after Tnni3 knock down through siRNA, *n* = 6. (D) Candidate genes mRNA expression detection after Tnni3 overexpression through Ad transfection. *n* = 6. *p < 0.05; **p < 0.01; ***p < 0.001; ****p < 0.0001. Values are expressed as mean ± SD.

Next, sequencing and bioinformatic analysis were used to explore the role of cTnI on cardiac autophagy. RNA‐sequencing data in cTnI knock‐out homozygous mouse heart suggested 178 differentially expressed genes when log_2_ fold change was set up as >1. In our previous studies, we found that cTnI might function as a co‐factor of YY1. We then analysed DEGs by overlapping them with YY1 ChIP‐sequencing data. A total number of 152 genes were filtered by overlapping with YY1 ChIP‐sequencing data (Figure 3A). The volcano diagram indicated that genes such as Egr1, FOS and FOSb were down‐regulated, and Gstp2, Lrp8 and Mmp9 were up‐regulated (Figure 3B, Table S2). Most of those genes' expression patterns were verified by cTnI gain and loss in vitro experiments, and FOS was selected as a candidate downstream gene of cTnI, as its expression patterns were consistent with data of cTnI gain and loss (Figure 3C,D).

### 3.4 FOS expression in ageing and cTnI ^+/−^ Hearts

**FIGURE 4:**
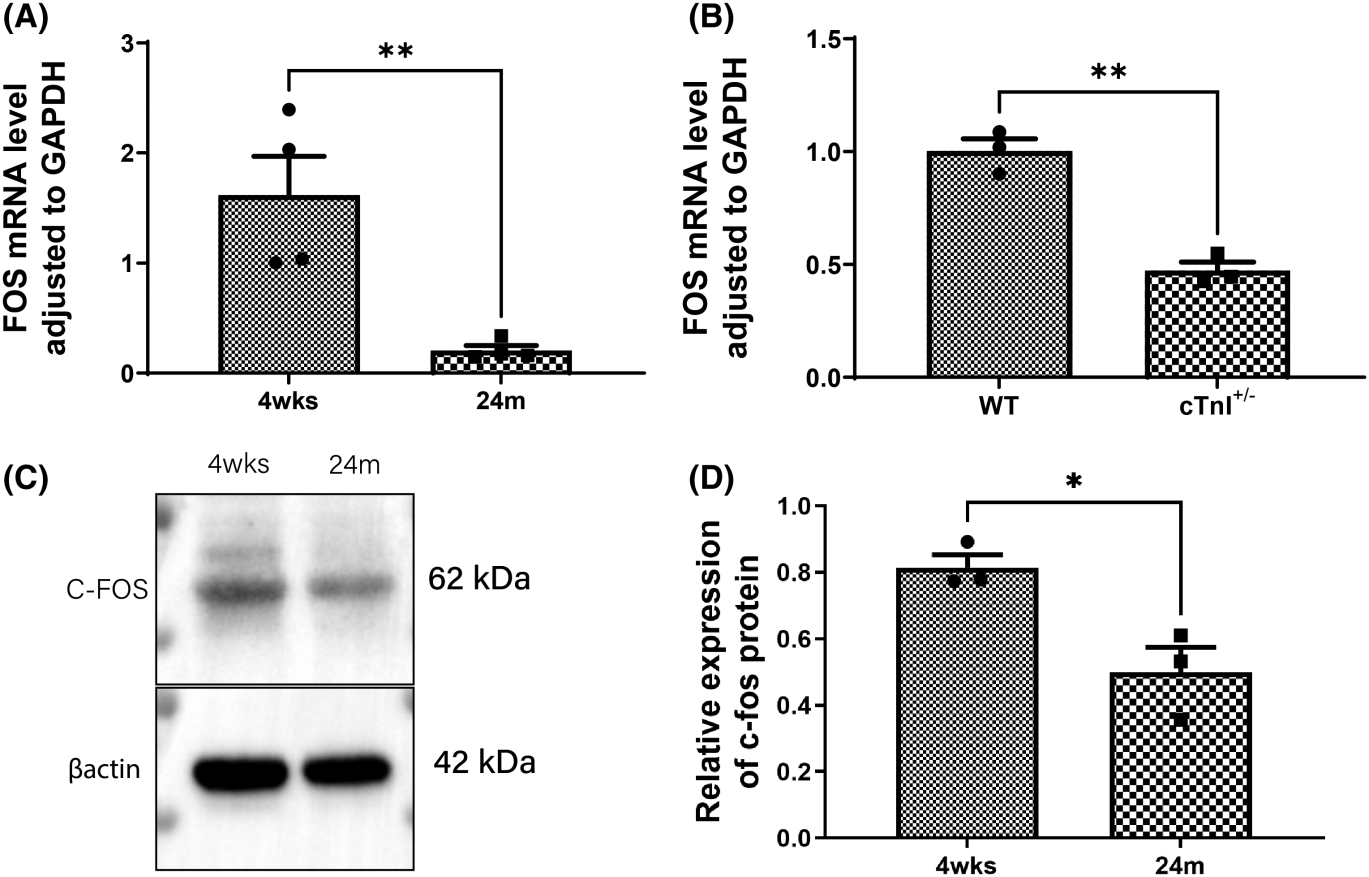
FOS expression in ageing and cTnI^+/−^ hearts. (A) qRT‐PCR of FOS mRNA levels in 4 weeks and 24 months mouse hearts, *n* = 4. (B) qRT‐PCR of FOS mRNA levels in 12‐month WT and cTnI^+/−^ mouse hearts, *n* = 3. (C, D) FOS protein, c‐fos levels in 4 weeks and 24 months mouse hearts, *n* = 3. *p < 0.05; **p < 0.01. Values are expressed as mean ± SD.

The above‐mentioned data strongly suggested that FOS might mediate the regulatory role of cTnI on cardiac ageing. We then detected FOS expression levels in ageing and cTnI^+/−^ hearts. FOS mRNA levels decreased significantly both in ageing and cTnI^+/−^ hearts (Figure 4A,B). c‐Fos, the protein encoded by FOS, was reduced in ageing cardiac tissues (Figure 4C,D).

### 3.5 cTnI binding affinity on FOS's promoter reduced in ageing heart

**FIGURE 5:**
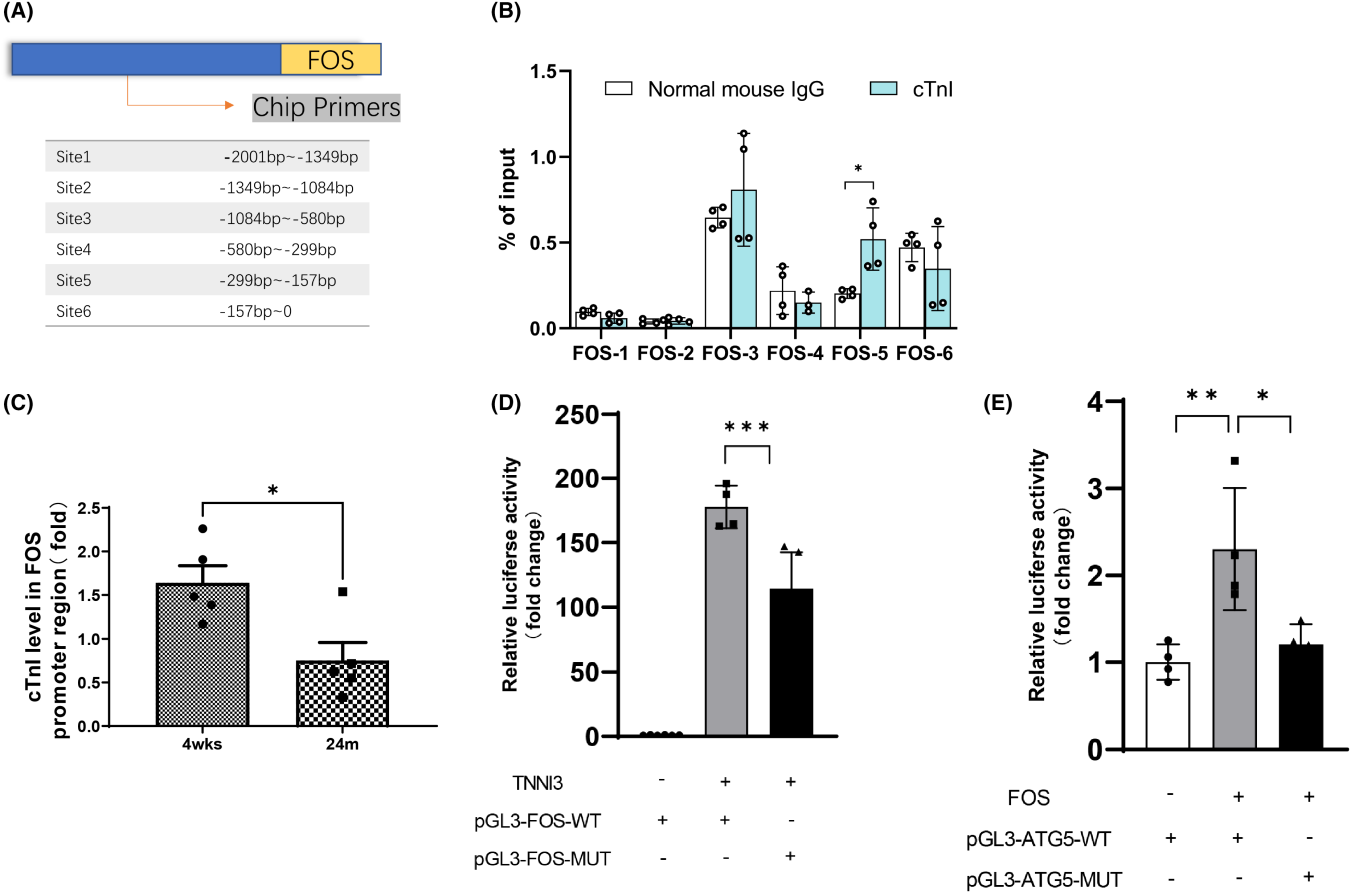
cTnI‐binding affinity on FOS's promoter reduced in ageing heart. (A) The promoter region of FOS was divided into six segments. (B) Binding abilities of each segment of cTnI with FOS promoter were verified by CHIP‐qPCR, *n* = 4. (C) ChIP‐q‐PCR detection of cTnI binding level in FOS promoter in mouse hearts of 4 weeks and 24 months, *n* = 5. (D) Relative luciferase activity of cells with co‐transfected of TNNI3 overexpression plasmid (TNNI3) and FOS promoter luciferase reporter gene plasmid (pGL3‐FOS‐WT) or FOS mutant promoter luciferase reporter gene plasmid (pGL3‐FOS‐MUT), *n* = 4. (E) Relative luciferase activity of cells co‐transfected with FOS overexpression plasmid (FOS) and ATG5 promoter luciferase reporter gene plasmid (pGL3‐FOS‐WT) or ATG5 mutant promoter luciferase reporter gene plasmid (pGL3‐ATG5‐MUT), *n* = 4. *p < 0.05, **p < 0.01, ***p < 0.001. Values are expressed as mean ± SD.

cTnI could bind with the ‘CCAT’ motif as a co‐factor of YY1, as proved in our previous study. Thus, binding levels of cTnI with FOS's promoter region were detected in the heart tissue by ChIP‐q‐PCR technology. Specific binding DNA sequences of cTnI were enriched in the FOS promoter −299 to −157 region (Site 5, Figure 5A,B). cTnI binding levels with the FOS promoter region declined significantly in ageing hearts (Figure 5C). In 293T cells, pcDNA3.1 plasmid /TNNI3 overexpression plasmid and FOS promoter luciferase reporter gene plasmid were co‐transfected. Compared with control group, the luciferase activities significantly increased in cells transfected with pcDNA3.1‐TNNI3 plasmid (Figure 5D). These data indicate that cTnI could regulate FOS expression by binding with its promoter region.

### 3.6 FOS could enhance transcriptional activity of ATG5

As a classic transcription factor, FOS has been reported as an important factor in multiple pathways. There are also studies that found FOS could regulate autophagy in Parkinson's disease by increasing Becn1 expression.^9^ Using the Jaspar website, ‘GTGAGCCA’ was predicted as a potential binding sequence of FOS on ATG5's promoter. In 293T cells, pcDNA3.1 plasmid /FOS overexpression plasmid and ATG5 promoter luciferase reporter gene plasmid were co‐transfected. Compared with control group, the luciferase activities significantly increased in cells transfected with pcDNA3.1‐FOS plasmid (Figure 5E).

## 4 DISCUSSION

In the present study, we provide more evidence that cTnI could be located in the nucleus and that the NLS of cTnI might be located on a segment of 16‐22aa. Moreover, decreased cTnI might down‐regulate FOS expression in ageing mouse hearts, accompanied by declined ATG5. FOS was proved to bind with the ATG5 promoter and was critical for reducing ATG5 transcription in ageing‐associated cardiac autophagy impairment.

Some studies have demonstrated that cTnI could be found in the nucleus,^11^, ^13^, ^17^ and our recently published paper^14^ showed that TnI is located in the nuclear of fetal human hearts. We also found that levels of cTnI nucleoprotein in the left ventricle are higher than in the right ventricle. Moreover, intranuclear cTnI proteins decline in the left ventricles of heart failure more than in controls.^14^ Therefore, we speculate that the content of intranuclear cTnI in cardiomyocytes may be associated with heart functions. In this study, we focus on cardiac ageing, accompanied by autophagy impairment. In ageing mouse cardiac tissue, intranuclear cTnI, ATG5 and cardiac autophagy levels decreased. Interestingly, cardiac autophagy levels declined in cTnI^+/−^ mouse hearts, as autophagy markers LC3B II and ATG5 reduced, and P62 increased. There were no statistical differences of ATG7 expression levels between young adult and ageing heart. ATG7‐related decreased autophagy appears to have a negative effect on the vascular system.^18^ Low levels of Atg7 have been reported in aged brain tissue.^19^ Our data indicate that intranuclear cTnI decrease is associated with ATG5‐related cardiac autophagy damage.

In our previous study,^14^ genome‐wide sequence motifs targeted by cTnI in heart tissues by using ChIP‐Seq analysis identified the sequence CCAT as cTnI's binding motif, which is also required for YY1‐binding to the promoter area of YY1‐related genes.^20^, ^21^ Moreover, the binding abilities of cTnI and YY1 were confirmed. So, we believed that intranuclear cTnI plays a regulatory role in cardiomyocytes as a co‐factor of YY1. Thus, RNA‐sequencing data of cTnI knockout heart tissues and YY1 ChIP‐sequencing data were weighted and analysed. Overlapping data indicated some genes associated with cardiac ageing, such as s100a8,^22^, ^23^ FOS^24^, ^25^ and FOSb.^26^ By cTnI gain and loss studies, FOS was filtered as a potential target of cTnI. FOS has been reported as an essential factor in BECN1‐induced autophagy in Parkinson's disease.^9^ Meanwhile, several studies have demonstrated that YY1 could regulate FOS expression.^27^, ^28^, ^29^ Using Jaspar webserver, potential binding sites of FOS/AP1 were found in the ATG5 promoter region. At last, dural luciferase report assay results indicated FOS could regulate ATG5 transcriptional activity. Our data indicated that intranuclear cardiac troponin I might be a marker or a therapeutic target on cardiac ageing.

Taken together, our data demonstrated that decreased intranuclear cTnI might impair cardiac autophagy through down‐regulating FOS/ATG5 pathway in ageing hearts. This reveals a novel understanding of intranuclear cTnI in the regulation of cardiac ageing, and may also provide some clues for the interventions on autophagy dysregulation in ageing hearts.

## AUTHOR CONTRIBUTIONS

**Rui Min Liu:** Data curation (equal); formal analysis (equal); methodology (equal). **Shan Huang:** Data curation (equal); investigation (equal); validation (equal). **Di Hu:** Software (equal); validation (equal). **Lingjuan Liu:** Project administration (equal); resources (equal); validation (equal). **Hui Chao Sun:** Formal analysis (equal); funding acquisition (equal). **Jie Tian:** Conceptualization (equal); funding acquisition (equal); writing – review and editing (equal). **Bo Pan:** Conceptualization (lead); funding acquisition (equal); investigation (lead); writing – original draft (lead); writing – review and editing (equal).

## FUNDING INFORMATION

This work was supported by the Natural Science Foundation of China (Grant Number: 81974030 to JT), Natural Science Foundation of CHONGQING (Grant Number: CSTB2023NSCQ‐MSX0077 to BP) and CHONGQING Science and Technology Foundation (Grant Number: cstc2019jcyj‐msxmX0866 to HCS).

## CONFLICT OF INTEREST STATEMENT

The authors declare no conflicts of interest.

## Supporting information


Figure S1

Table S1

Table S2

## Data Availability

All relevant data and materials are stored at Children's Hospital of Chongqing Medical University and can be obtained from the first author and corresponding author.
