# Supplementary material for: Decreased intranuclear cardiac troponin I impairs cardiac autophagy through FOS/ATG5 in ageing hearts
Source: J Cell Mol Med. 2024 Apr 29;28(9):e18357. doi: 10.1111/jcmm.18357 (PMC11057418; doi:10.1111/jcmm.18357)
Supplement: Supplementary file 1 — Figure S1 [file JCMM-28-e18357-s002.docx]

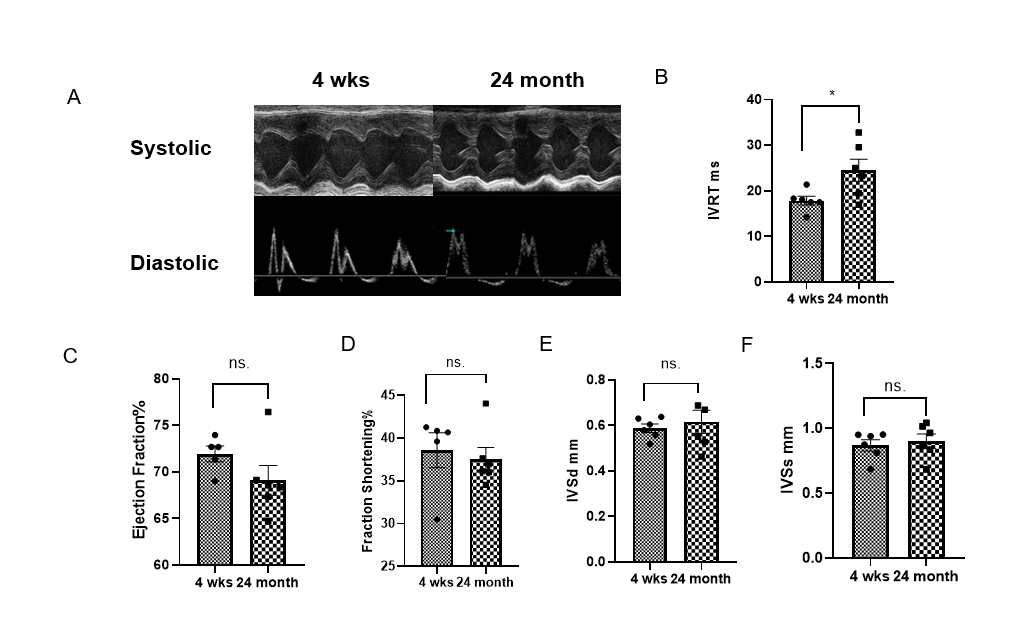


**Figure S1 Diastolic Function Decreases at 24-Months-Mice Hearts**

**(A)**Representative systolic and diastolic echocardiography images of 4-week and 24-month mice. **(B-F)** Left ventricular function of 4-week and 24-month mice, assessed by Left ventricular ejection fraction (LVEF), fractional shortening (FS), Interventricular septal thickness at diastolic (IVS;d); Interventricular septal thickness at systolic (IVS;s), **p* < 0.05, ns. means no significance; 4-week vs 24-month, n=6.
