# Supplementary material for: Decreased intranuclear cardiac troponin I impairs cardiac autophagy through FOS/ATG5 in ageing hearts
Source: J Cell Mol Med. 2024 Apr 29;28(9):e18357. doi: 10.1111/jcmm.18357 (PMC11057418; doi:10.1111/jcmm.18357)
Supplement: Supplementary file 2 — Table S1 [file JCMM-28-e18357-s003.pdf]

qPCR primers

**Gene**	**sequence**	**product（bp）**
GAPDH Forward	5‘-AAGAAGGTGGTGAAGCAGGCATC-3’	118
GAPDH Reverse	5‘-CGGCATCGAAGGTGGAAGAGTG-3’	118
TNNI3 Forward	5‘-AGGAGATGGAACGAGAGGCAGAAG-3’	129
TNNI3 Reverse	5‘-CGTGAAGCTGTCGGCATAAGTCC-3’	129
S100a8 Forward	5‘-GGAGTTCCTTGCGATGGTGA-3’	78
S100a8 Reverse	5‘-GGCCAGAAGCTCTGCTACTC-3’	78
S100a9 Forward	5‘-CTGCATGAGAACAACCCA-3’	125
S100a9 Reverse	5‘-AGGCCATTGAGTAAGCCA-3’	125
Lrp8 Forward	5‘-CCGGCAAACACTGGTTTCAG-3’	592
Lrp8 Reverse	5‘-CTGGTAGGTAGGGTCGTGGA-3’	592
Egr1 Forward	5‘-AGTCGGGCTCCCAGGACTTA-3’	326
Egr1 Reverse	5‘-GGCAAACTTCCTCCCACAAAT-3’	326
Fos Forward	5‘-TTTATCCCCACGGTGACAGC-3’	598
Fos Reverse	5‘-TGCTCTTGACTGGCTCCAAG-3’	598
Fosb Forward	5‘-GTGTAGCTCATCACCCTCCG-3’	507
Fosb Reverse	5‘-AGTCGATCTGTCAGCTCCCT-3’	507
Mmp9 Forward	5‘-CCGCGAGACATGATCGATGA-3’	521
Mmp9 Reverse	5‘-CCTTTAGTGGTGCAGGCAGA-3’	521
Gstp2 Forward	5‘-CTACTTCCCAAGTCCAGGGC-3’	590
Gstp2 Reverse	5‘-TGGGACGGTTCACATGTTCC-3’	590
Nr4a1 Forward	5‘-TCCCCGAGCCAGACTTATGA-3’	587
Nr4a1 Reverse	5‘-GGCTGCTTGGGTTTTGAAGG-3’	587
