# Supplementary material for: Decreased intranuclear cardiac troponin I impairs cardiac autophagy through FOS/ATG5 in ageing hearts
Source: J Cell Mol Med. 2024 Apr 29;28(9):e18357. doi: 10.1111/jcmm.18357 (PMC11057418; doi:10.1111/jcmm.18357)
Supplement: Supplementary file 3 — Table S2 [file JCMM-28-e18357-s001.pdf]

gene_nam	log2FoldC	pvalue	padj
Tnni3	-14.2462	3.2E-153	4.6E-149
S100a8	2.355363	9.76E-19	7.03E-15
Rtn4r	-3.03448	1.03E-14	4.96E-11
Cav1	-1.17609	3.11E-14	9.08E-11
Lrp8	2.438065	3.15E-14	9.08E-11
Eps8l1	-3.61201	2.73E-12	6.56E-09
S100a9	2.048376	1.79E-11	3.22E-08
Egr1	-2.30673	9.24E-11	1.33E-07
Ifitm6	2.788911	1.37E-10	1.79E-07
Stfa3	3.559508	1.84E-10	2.21E-07
Gimap4	-1.13221	1.15E-09	1.19E-06
Synpo2l	1.214098	3.17E-09	2.85E-06
Nppa	1.676714	9.14E-09	7.75E-06
Ifi203	-1.51883	1.16E-08	9.25E-06
Rasgrp3	-1.10417	1.81E-08	1.38E-05
Ccdc8	1.311213	3.61E-08	2.48E-05
Adamts8	1.657364	5.71E-08	3.74E-05
Lcn2	1.677997	6.29E-08	3.94E-05
Efnb3	1.657092	7.55E-08	4.35E-05
Ppp1r16b	-1.29426	8.42E-08	4.66E-05
Cilp2	-2.24317	1.2E-07	6.2E-05
S1pr3	-1.1989	2.73E-07	0.000136
Akr1c14	-1.5052	3.48E-07	0.000167
Tspan13	-1.0225	4.19E-07	0.000189
Srgn	-1.10112	7.44E-07	0.000325
Gpr157	-1.0569	8.99E-07	0.000366
Tnfrsf12a	1.39064	9.14E-07	0.000366
Stfa2	2.407692	9.99E-07	0.000389
Scgb1a1	-21.4285	1.17E-06	0.000425
Myadm	-1.10888	1.21E-06	0.000425
Capn11	-21.399	1.21E-06	0.000425
Sfrp2	1.164023	1.24E-06	0.000425
Hspb7	1.020764	1.49E-06	0.00047
Art1	-1.11625	1.51E-06	0.00047
Tnnt3	3.004092	1.53E-06	0.00047
Cd177	2.834109	2.2E-06	0.000597
St8sia4	-1.39181	2.7E-06	0.000695
Fosb	-2.45942	2.87E-06	0.000705
Kctd12b	-1.88866	2.89E-06	0.000705
Smim5	1.147719	3.01E-06	0.000722
Scgb3a2	-20.5478	3.15E-06	0.00073
Prdm1	-2.3339	3.17E-06	0.00073
Nts	-2.19034	3.19E-06	0.00073
Slc25a25	-1.91015	3.43E-06	0.000771
Retnlg	1.81051	3.56E-06	0.000787
Edn1	1.080073	3.95E-06	0.000837
Cdc42ep2	-1.03168	5.17E-06	0.001049
Il2rg	-1.56544	5.99E-06	0.001136
Armc2	-2.08146	6.13E-06	0.001146
Gpr4	-1.15907	6.25E-06	0.001155
Ifi27l2a	-1.28008	6.4E-06	0.001167
Mmp9	2.237935	6.86E-06	0.001236
Spsb4	1.245244	7.82E-06	0.001391
Kcnf1	6.389371	8.9E-06	0.001509
Nt5e	1.099671	9.49E-06	0.001591
Mr1	1.005062	1.23E-05	0.001966
Masp1	1.219214	1.3E-05	0.002039

Ltf	2.491225	1.61E-05	0.002417
Sez6l2	-1.43688	1.63E-05	0.002417
Olfr1396	-1.65036	1.7E-05	0.002498
Alad	-1.13389	2.12E-05	0.003051
Irf7	-1.33397	2.14E-05	0.003055
Frem2	1.096875	2.2E-05	0.003108
Stfa2l1	2.47123	2.39E-05	0.003316
Clu	1.039831	2.64E-05	0.003567
Aplnr	-1.18365	2.65E-05	0.003567
Pirt	1.16874	2.9E-05	0.003778
Tent5b	1.708058	2.91E-05	0.003778
Gstp2	2.198359	3.01E-05	0.003796
Myh7	5.10344	3.02E-05	0.003796
Usp11	1.271275	3.15E-05	0.003853
Ier2	-1.24794	3.18E-05	0.003853
Asprv1	2.273739	3.27E-05	0.00389
Adra1b	1.099005	3.36E-05	0.003971
Gimap6	-1.0698	3.59E-05	0.004133
Arhgap18	-1.01945	3.61E-05	0.004133
Pcdh17	-1.1364	4.41E-05	0.004823
Gfpt2	-1.1029	4.46E-05	0.004823
Ndufa4l2	-1.04175	4.46E-05	0.004823
Lgals3bp	-1.08221	4.53E-05	0.004823
Cd248	-1.18383	4.57E-05	0.004823
Plk3	-1.81682	4.58E-05	0.004823
Dusp5	-1.64308	4.72E-05	0.004925
Ifi44	-1.95709	4.86E-05	0.004953
Camk1d	1.183573	4.87E-05	0.004953
Optn	-1.06549	4.98E-05	0.004985
Stfa1	2.159601	5.1E-05	0.005059
Galm	-1.08773	5.16E-05	0.00506
Bcl6b	-1.36226	5.56E-05	0.00534
Tlnrd1	-1.06053	6.23E-05	0.005718
Mt1	-1.10165	7.03E-05	0.006295
Spry4	-1.31987	8.49E-05	0.007324
Meox2	-1.26141	0.000107	0.008875
Tox3	1.085323	0.000111	0.009136
Camk1g	1.406452	0.000131	0.010137
Ifit1	-1.57584	0.000134	0.010265
Fgf12	1.156077	0.000142	0.010678
Nuak1	1.155785	0.000147	0.010906
Tnni1	3.694102	0.000186	0.013358
Podxl2	1.060666	0.000188	0.013405
Ctla2a	-1.31037	0.000199	0.013955
Cpt1b	1.49063	0.000205	0.01423
Slfn4	1.554265	0.000209	0.014301
Gbp2	-1.02807	0.000242	0.015481
Pdgfb	-1.2216	0.000242	0.015481
Apln	-1.48436	0.000247	0.01567
Kcnv2	-1.85513	0.000253	0.01584
Tppp3	-1.17386	0.000256	0.015893
Nrarp	-1.90045	0.000281	0.017026
Camp	2.400671	0.000301	0.018174
Dnah8	-2.47647	0.000313	0.018719
Nos2	-1.25603	0.000332	0.019668
Fbn2	1.289643	0.000334	0.019749
Cyfip2	-1.09472	0.000348	0.020215
Herc3	1.275594	0.000349	0.020215

Csf2rb	1.260726	0.000349	0.020215
C530008N	1.166799	0.000352	0.020264
Plekho2	-1.01787	0.000363	0.020864
Hlx	-1.3305	0.000415	0.02232
AU021092	-1.08228	0.000415	0.02232
Nepn	-1.88212	0.000431	0.022918
Fmr1nb	3.368181	0.000447	0.023354
Fos	-3.58984	0.000449	0.023354
Sphk1	1.235953	0.000456	0.023655
Ankrd1	1.131334	0.000462	0.023799
Kcnmb1	-1.70171	0.0005	0.024998
Adam33	1.684793	0.000512	0.025431
Ppfia4	1.159125	0.000541	0.026348
Wfikkn2	1.710251	0.00056	0.027091
Adora1	1.282662	0.000576	0.027653
Kcna4	1.299212	0.000589	0.028203
H2-T24	-1.13862	0.000605	0.028728
Jun	-1.17688	0.00064	0.029793
Fgfbp3	1.258313	0.000666	0.030762
Snca	-1.3541	0.00075	0.033895
Phf11d	-1.07109	0.00075	0.033895
Tox	1.845111	0.000773	0.0348
Cytl1	-1.29885	0.000838	0.037366
Prune2	-1.1533	0.000923	0.040294
Ramp3	-1.39791	0.000963	0.041615
Scin	1.307236	0.000983	0.04212
Spin4	-1.41494	0.000989	0.04212
Prkag3	1.825307	0.001004	0.04212
Bhlhe40	-1.18226	0.001047	0.043165
Mmp8	1.900653	0.001128	0.04579
Nr4a1	-3.35537	0.001129	0.04579
Chil3	1.863987	0.001204	0.047875
Atg9b	-1.8528	0.001205	0.047875
Arc	-3.56939	0.001237	0.048589
Stk32b	1.863464	0.00126	0.048954
Apold1	-3.44004	0.001291	0.049844
Shisa4	1.230992	0.001293	0.049844
